# Toll-like receptor 4 mediates Lewis lung carcinoma-induced muscle wasting via coordinate activation of protein degradation pathways

**DOI:** 10.1038/s41598-017-02347-2

**Published:** 2017-05-23

**Authors:** Guohua Zhang, Zhelong Liu, Hui Ding, Hongyu Miao, Jose M. Garcia, Yi-Ping Li

**Affiliations:** 1 Department of Integrative Biology and Pharmacology, University of Texas Health Science Center at Houston (UTHealth), Houston, 77030 Texas USA; 2 Division of Endocrinology, Tongji Hospital, Tongji Medical College, Huazhong University of Science and Technology, Wuhan, China; 3 Department of Respiratory Medicine, Yixing Hospital affiliated to Jiangsu University, Yixing, China; 4 School of Public Health, The University of Texas Health Science Center at Houston (UTHealth), Houston, Texas USA; 5 Geriatric Research, Education and Clinical Center (GRECC), VA Puget Sound Health Care System, University of Washington, Seattle, 98108 WA USA

## Abstract

Cancer-induced cachexia, characterized by muscle wasting, is a lethal metabolic syndrome with undefined etiology. Current consensus is that multiple factors contribute to cancer-induced muscle wasting, and therefore therapy requires combinational strategies. Here, we show that Toll-like receptor 4 (TLR4) mediates cancer-induced muscle wasting by directly activating muscle catabolism as well as stimulating an innate immune response in mice bearing Lewis lung carcinoma (LLC), and targeting TLR4 alone effectively abrogate muscle wasting. Utilizing specific siRNAs we observed that LLC cell-conditioned medium (LCM)-treated C2C12 myotubes underwent a rapid catabolic response in a TLR4-dependent manner, including activation of the p38 MAPK−C/EBPβ signaling pathway as well as the ubiquitin-proteasome and autophagy-lysosome pathways, resulting in myotube atrophy. Utilizing a reporter cell-line it was confirmed that LCM activated TLR4. These results suggest that LLC-released cachexins directly activate muscle catabolism via activating TLR4 on muscle cells independent of immune responses. Critically, LLC tumor-bearing TLR4^−/−^ mice were spared from muscle wasting due to a blockade in muscle catabolic pathways. Further, tumor-induced elevation of circulating TNFα and interleukin-6 (IL-6) was abolished in TLR4^−/−^ mice. These data suggest that TLR4 is a central mediator and therapeutic target of cancer-induced muscle wasting.

## Introduction

Cancer-induced cachexia, or cancer cachexia, is a highly complex metabolic syndrome characterized by progressive muscle wasting. Prominent clinical features of cachexia are weight loss, inflammation, insulin resistance, and increased muscle protein breakdown. About half of all cancer patients develop cachexia, and cachexia is directly responsible for about 30% of all cancer-related deaths^1^. The precise etiology of cancer cachexia is unknown. The current consensus is that multiple factors contribute to cancer cachexia, and therapy requires combinational strategies^2, 3^.

A key feature of cancer cachexia is systemic inflammation and anti-inflammation strategies are considered central to the therapy of cancer cachexia. Multiple inflammatory cytokines such as TNFα, interleukin-6 (IL-6) and interleukin-1 (IL-1) are elevated in cachectic cancer hosts and are known to promote muscle catabolism, thus are considered therapeutic targets^4^. Cancer-induced muscle catabolism involves activation of proteolysis mediated by the ubiquitin-proteasome pathway (UPP)^5^ in muscle cells through cytokine-activated signaling molecules including NF-κB^6, 7^, p38 MAPK^8–10^ or JAK-STAT3 pathways^9, 11^. In addition, another major cellular proteolytic system, the autophagy-lysosome pathway (ALP), may also contribute to muscle wasting in animal models of cancer^12–14^ and cancer patients^15–17^. However, the underlying signaling mechanism that mediates cancer-induced activation of ALP is unknown.

The involvement of multiple humoral factors and intracellular signaling pathways in cancer-induced muscle wasting makes it difficult to intervene effectively in cancer-induced muscle wasting. However, we reasoned that if we could identify the origin of systemic inflammation in cancer hosts, effective therapy might be achieved by targeting the potential “master mediator” of inflammation. Although certain types of cancer can release cytokines, the bulk of circulating cytokines in cancer hosts appears released by host tissues as a response to cancer^18^. Cancer can generate danger-associated molecular patterns (DAMPs) that activate TLR4^19, 20^, a plasma membrane receptor that plays a central role in innate immunity^21^. Previously Cannon *et al*. observed that mice with nonfunctional TLR4 due to a TLR4 double mutation (TLR4^d/d^) were resistant to cachexia induced by squamous cell carcinoma SCCF VII cells, and suggested that TLR4 mediated muscle wasting by increasing circulating IL-1β^22^. However, the detailed mechanism through which TLR4 mediates cancer-induced muscle wasting remains unknown. Given that TLR4 is expressed by skeletal muscle cells^23, 24^, it is possible that cancer directly stimulates muscle catabolism by activating TLR4 on muscle cells. In addition, cancer-induced activation of TLR4 on immune cells would increase their synthesis and release of inflammatory cytokines that promote muscle catabolism. Thus, we tested the hypothesis that TLR4 is a key mediator of cancer-induced muscle wasting due to its integration of catabolic signaling through activating muscle protein degradation pathways directly and increasing cytokine release indirectly. Utilizing *in vitro* as well as *in vivo* approaches, we found evidence in the present study that supports our hypothesis. Our data suggest that TLR4 is a central mediator of cancer-induced muscle protein degradation through the UPP and the ALP, and thus may be a key therapeutic target of cancer cachexia.

## Results

### LLC cell-conditioned medium activates protein degradation pathways in C2C12 myotubes through TLR4

**Figure 1:**
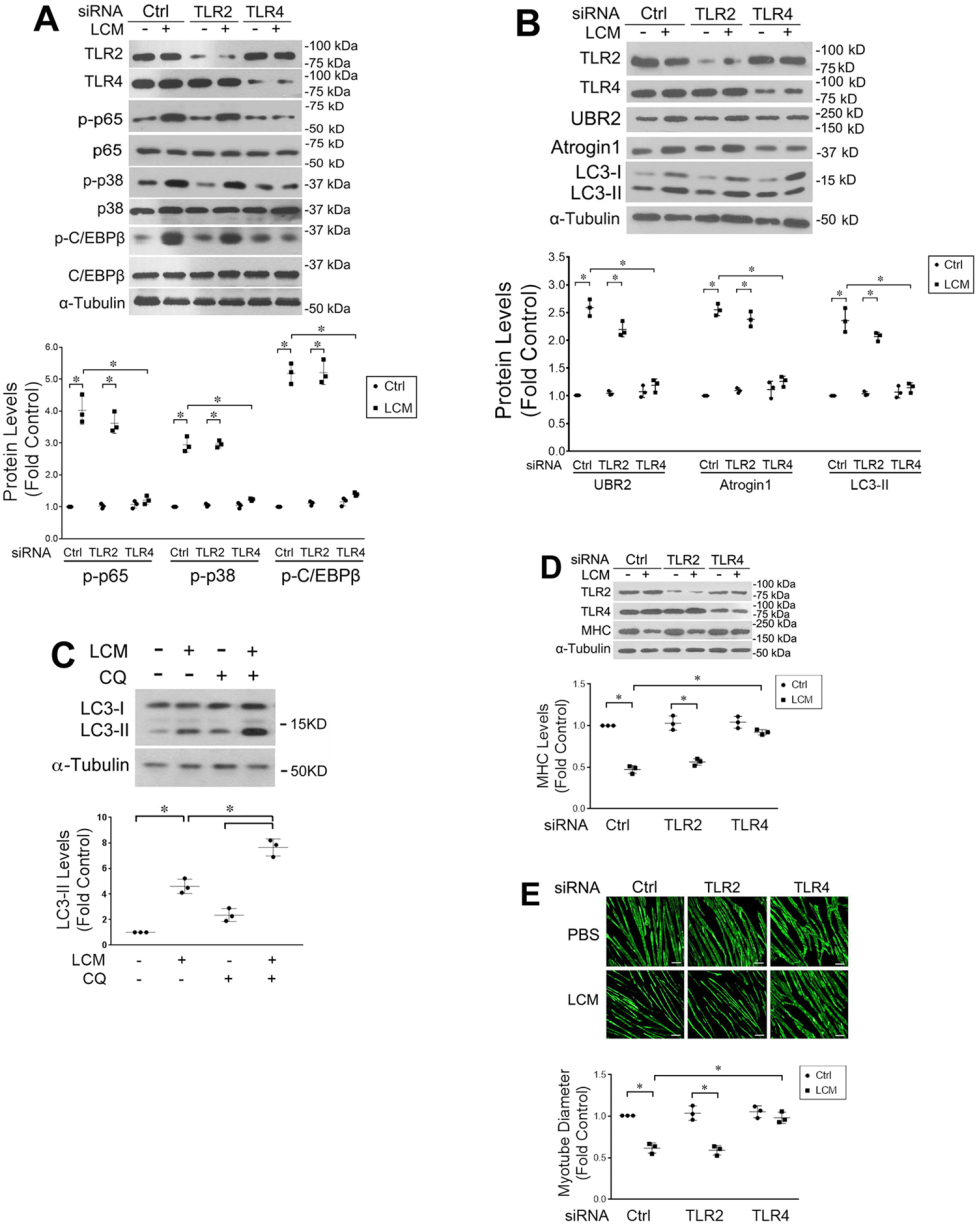
LLC cell-conditioned medium activates myotube catabolism through TLR4. C2C12 myoblasts were transfected with indicated siRNAs. After incubation in differentiation medium for 96 h, myotubes were treated with Lewis lung carcinoma cell-conditioned medium (LCM) or control medium. Activation of NF-κB (p65), p38 MAPK and C/EBPβ was evaluated by Western blot analysis of cell lysate in 1 h (**A**). Levels of UBR2, atrogin1 and LC3-II were evaluated by Western blot analysis of cell lysate in 8 h (**B**). Autophagy flux increase was demonstrated by measuring LC3-II levels in myotubes pre-treated with 20 μM of lysosome inhibitor chloroquine (CQ, **C**). Levels of myosin heave chain (MHC) were evaluated by Western blot analysis of cell lysate in 72 h (**D**). TLR2 and TLR4 knockdown was monitored at each of the time points. Myotubes treated for 72 h were immunofluorescence-stained for MHC and their diameter was measured (**E**). Data was analyzed by ANOVA. *Denotes a difference (*P* < 0.05).

**Figure 2:**
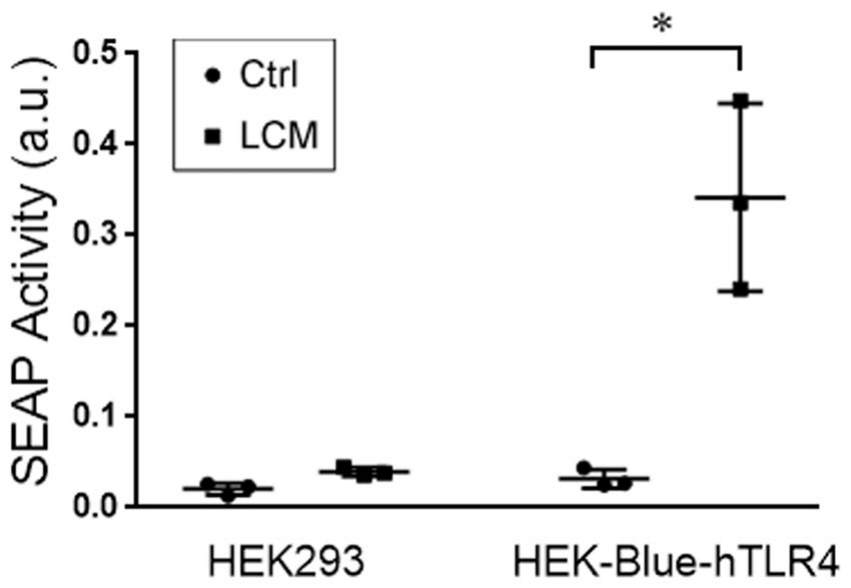
LLC cell-conditioned medium activates TLR4 in reporter cells. TLR4 reporter cell line HEK-Blue hTLR4 (InvivoGen) was treated with LCM or control medium for 24 h and TLR4 activation was measured as the enzymatic activity of secreted embryonic alkaline phosphatase (SEAP) in arbitrary unit (a.u.). Data was analyzed by Student *t* test. *Denotes a difference (*P* < 0.05).

We previously showed that treating cultured myotubes with LLC cell-conditioned medium (LCM) recapitulates the muscle catabolism seen in LLC tumor-bearing mice through the activation of the p38 MAPK-C/EBPβ signaling pathway which upregulates E3 ubiquitin ligases atrogin1^8^ and UBR2^25^. In addition, LCM activates autophagy in cultured myotubes^13^. These data indicate a direct activation of muscle catabolism by cancer-released cachexins independent of host response. However, the membrane receptor(s) through which LCM activates UPP- and ALP-mediated muscle catabolism was unknown. To test the hypothesis that the catabolic effect of LCM on muscle cells is specifically mediated by TLR4, we knocked down TLR4 or TLR2 in C2C12 myotubes using specific siRNAs. LCM treatment of myotubes transfected with scrambled siRNA (control) activated downstream effectors of TLR4, p38 MAPK (phosphorylation of T181/Y182) and NF-κB (phosphorylation of p65), as expected. In addition, we observed activation of transcription factor C/EBPβ (phosphorylation of T188) in 1 h (Fig. 1A), upregulation of ubiquitin ligases UBR2 and atrogin1, and activation of autophagy (indicated by increased LC3-II) in 8 h (Fig. 1B). In addition, by treating myotubes with lysosome inhibitor chloroquine we observed a further increase in LC3-II, indicating that LCM increased autophagy flux (Fig. 1C). Activation/upregulation of these catabolic mediators resulted in myofibrillar protein myosin heavy chain (MHC) loss (Fig. 1D) as well as myotube atrophy (Fig. 1E) in 72 h. TLR4 knockdown in myotubes abolished all the catabolic response to LCM and preserved myotube mass. In contrast, TLR2 knockdown did not alter the catabolic effect of LCM (Fig. 1). To verify whether LCM actually activated TLR4, a TLR4 reporter cell-line, HEK293 cells that overexpress TLR4 (HEK-Blue hTLR4, InvivoGen), was treated with LCM and resulted in activation of TLR4-controlled reporter gene, secreted embryonic alkaline phosphatase (SEAP, Fig. 2). Thus, LCM induces muscle catabolism through direct activation of TLR4 on muscle cells.

### TLR4 is critical to LLC tumor-induced muscle catabolism in mice

**Figure 3:**
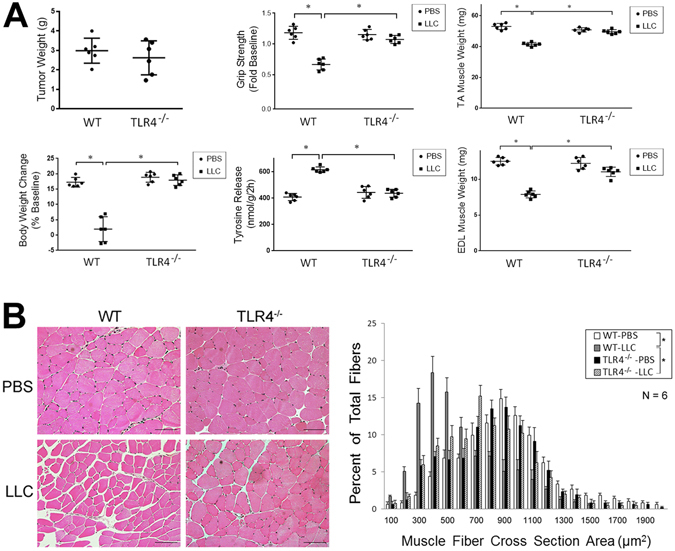
TLR4^−/−^ mice are resistant to LLC tumor-induced muscle wasting. Wild-type C57BL/6 and TLR4^−/−^ mice (7-week old male) were inoculated with LLC cells. Muscle wasting was evaluated in 21 days by measuring tumor mass, body weight change (excluding tumor weight), grip strength, tyrosine release from EDL, muscle weight (TA and EDL) (**A**) as well as muscle fiber cross-sectional area (**B**). Bar = 100 μm. Data was analyzed by ANOVA (**A**) or Chi-square analysis (**B**). *Denotes a difference (*P* < 0.05).

**Figure 4:**
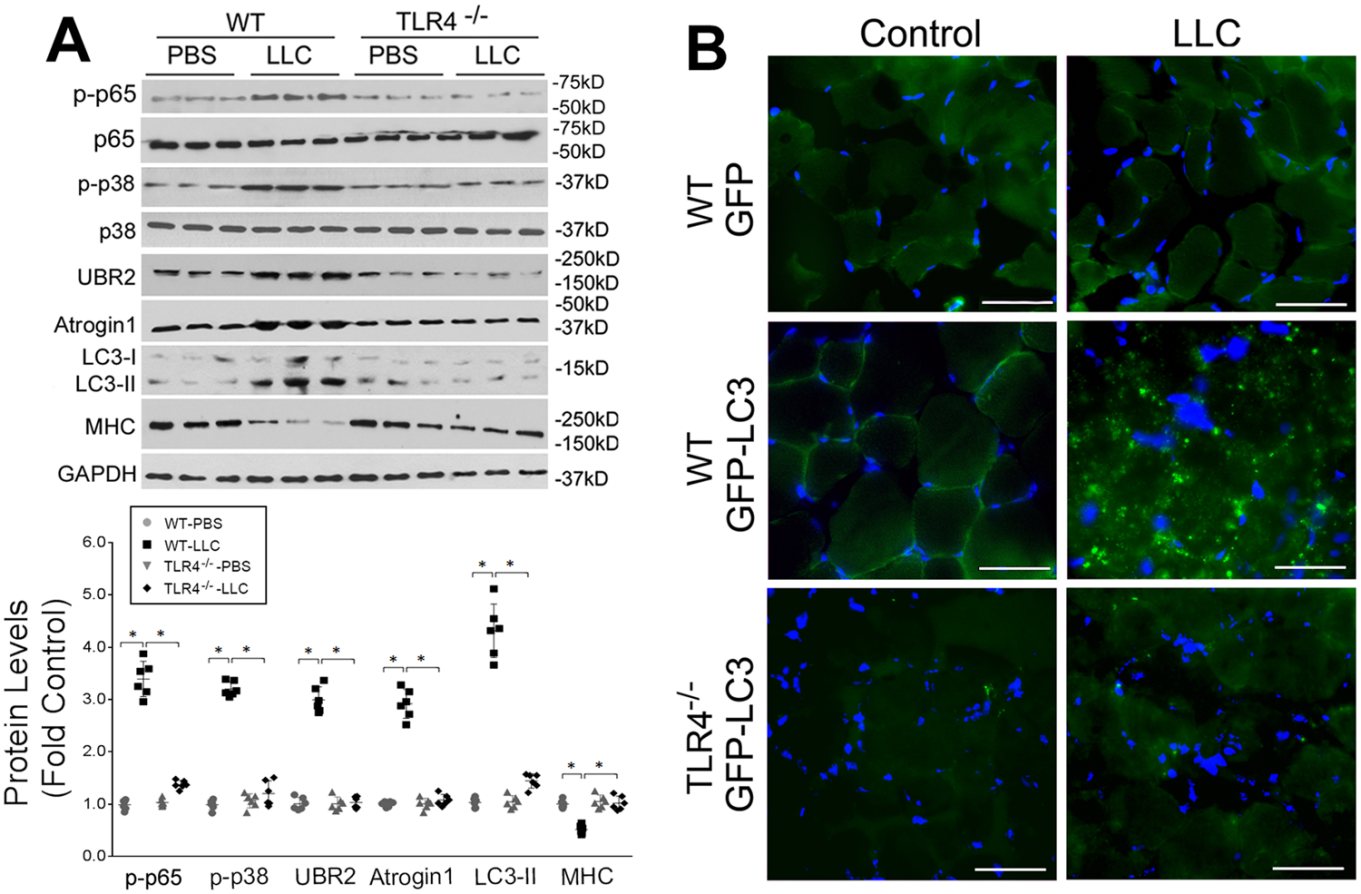
LLC tumor-induced muscle catabolism is dependent on TLR4. (**A**) TLR4 is required for the activation of catabolic pathways in muscle of LLC-bearing mice. TA muscle collected from mice described in Fig. 3 was analyzed by Western blotting for markers of TLR4-mediated signaling and muscle catabolism. Data was analyzed by ANOVA. *Denotes a difference (*P* < 0.05). (**B**) Autophagosome formation is activated in cachectic muscle of LLC-bearing mice in a TLR4-dependent manner. The TA of wild type C57BL/6 or TLR4^−/−^ mice that had been implanted with LLC cells (controlled by PBS injection) were transfected with GFP-LC3 or GFP-expressing plasmid on day 14. In 7 days, autophagosome formation was evaluated by fluorescence microscopy examination of frozen sections of TA. Bars represent 50 μM.

To determine whether LLC tumor induces muscle wasting *in vivo* through activation of TLR4-mediated protein degradation, we utilized the existing TLR4^−/−^ mice^26^ that were in the same C57BL/6 background as LLC to examine the role of TLR4 in cancer cachexia. In 21 days of LLC cell inoculation tumor-bearing wild-type mice developed muscle wasting as measured by changes in body and muscle mass, muscle strength (grip strength) and muscle proteolysis (tyrosine release from EDL muscle). However, TLR4^−/−^ mice were spared from muscle wasting, without altered tumor growth (Fig. 3A). Consequently, muscle fibers in tumor-bearing TLR4^−/−^ mice maintained normal histology and mass as measured by cross sectional area in contrast to tumor-bearing wild-type mice in which muscle fibers shrank and interstitial space increased (Fig. 3B). Further analysis of the activity of the catabolic pathways revealed that TLR4 deficiency prevented tumor-induced activation of p38 MAPK and NF-κB (p65), upregulation of ubiquitin ligases UBR2 and atrogin1, activation of autophagy, and loss of MHC (Fig. 4A). These results were similar to the response of TLR4-deficient myotubes to LCM described above. To verify whether autophagosome formation was increased in cachectic muscle of LLC tumor-bearing mice in a TLR4-dependent manner, we overexpressed GFP-LC3 in mouse TA and observed increased GFP-LC3-labeled autophagosomes in LLC tumor-bearing WT but not TLR4^−/−^ mice (Fig. 4B). These data suggest that TLR4 is critical to cancer-induced muscle wasting due to its mediation of muscle protein degradation through the UPP and ALP.

### TLR4 mediates LLC tumor-induced increase in circulating cytokines

**Figure 5:**
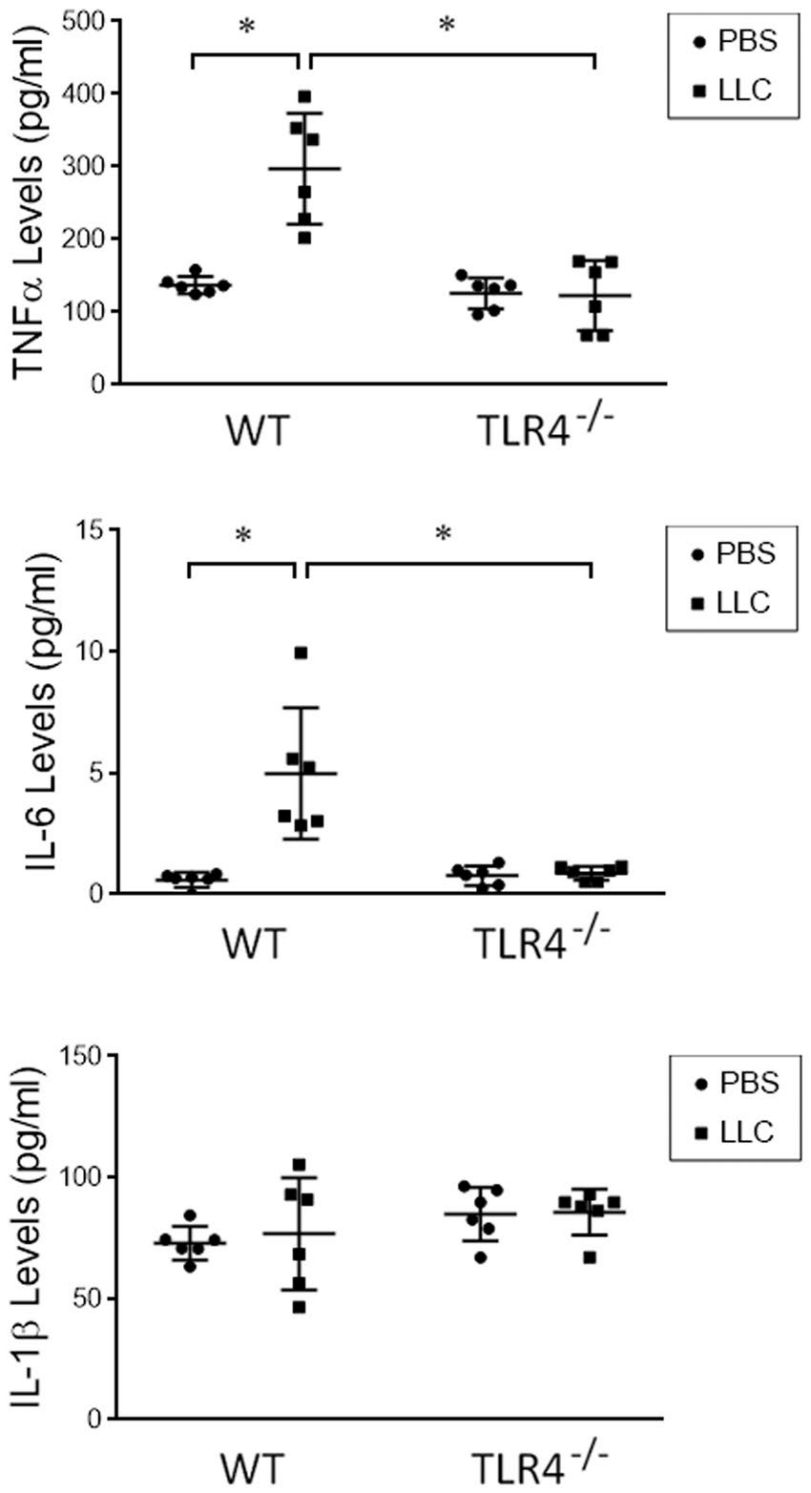
TLR4 is required for LLC tumor-induced elevation of circulating catabolic cytokines in mice. Sera collected from mice described in Fig. 3 on day 21 of LLC tumor implant were analyzed for catabolic cytokines TNFα, IL-6 and IL-1β by multiplex immunoassay (Bio-Rad). Data was analyzed by ANOVA. *Denotes a difference (*P* < 0.05).

Systemic activation of TLR4 increases cytokine synthesis and release from various host cells as an innate immune response^21^. We postulated that TLR4 knockout prevents the increase in circulating cytokines that could stimulate muscle catabolism such as TNFα, IL-6 and IL-1β, and determined the effect of TLR4 deficiency on circulating cytokines in LLC tumor-bearing mice. We found that serum TNFα and IL-6 levels were elevated in wild-type LLC tumor-bearing mice that developed cachexia, while IL-1β level remained unchanged. However, TNFα and IL-6 levels did not increase in TLR4^−/−^ tumor-bearing mice (Fig. 5). These data suggest that TLR4 plays a central role in mediating tumor-induced muscle wasting by activating muscle catabolism directly as well as by increasing the release of inflammatory cytokines to enhance muscle catabolism indirectly. Thus, perturbing TLR4 signaling systemically may prevent muscle wasting by abrogating both its direct and indirect effects.

## Discussion

The current study reveals that TLR4 is a central mediator of muscle catabolism in a mouse lung cancer model *in vitro and in vivo* due to its coordinate activation of the UPP and the ALP. Importantly, our data demonstrate that LLC directly activates muscle catabolism through the activation of TLR4 on muscle cells independent of host input. In addition, elevation of circulating TNFα and IL-6 in LLC tumor-bearing mice is dependent on TLR4. These data suggest that tumor-induced activation of TLR4 is responsible for both the systemic inflammation and muscle catabolism. These observations explain why previous clinical trials using anti-cytokine strategies had only limited success on intervening muscle wasting in cancer patients^27–29^ and suggest anti-TLR4 strategies could achieve much better results. Our findings open the door to a new therapeutic strategy that targets only a single plasma membrane receptor to alleviate systemic inflammation and abrogate muscle wasting in cancer hosts. Thus, cancer cachexia may become manageable by using a single reagent that perturbs TLR4 signaling.

Observed dependence of LCM-induced myotube catabolism on TLR4 and the rapid activation of TLR4 downstream effector p38 MAPK within 1 h indicate that LLC directly activates muscle catabolism without the need for new protein synthesis/release by host tissues. Thus, cancer-released cachexins instead of host responses to cancer are likely the primary cause of cancer-induced muscle wasting. However, the identity of LLC-released cachexin(s) that activates TLR4 is currently unknown. Identification of the cachexin(s) responsible for TLR4 activation is therefore warranted.

Our data suggest that TLR4 is a specific mediator of cancer-induced muscle catabolism. Despite the fact that LLC activates TLR2 via releasing versican^18^ and muscle cells express TLR2^23, 24^, our data indicates that TLR2 is not responsible for the catabolic effect of LLC on muscle. The difference between the intracellular signaling mechanism of TLR2 and TLR4 is that TLR2 signaling is dependent on MyD88 while TLR4 signaling involves TRIF in addition to MyD88^21^. Thus, the catabolic effect of LLC is likely being mediated by TRIF, which is consistent with the previous observations that MyD88 does not mediate LLC or LPS-induced muscle wasting^30^. On the other hand, TLR7 that also relies on MyD88 for signaling mediates muscle cell death in cancer cachexia^31^.

Recent clinical studies of cancer cachexia patients showed diverse patterns of increase in circulating cytokines. TNFα was most consistently increased in these patient populations, followed by IL-6, with IL-1β being less consistently increased^32, 33^. Our finding on the increase of serum TNFα and IL-6, but not IL-1β, in LLC tumor-bearing mice is similar to these patient data. On the other hand, whether cytokine increase in cancer patients is dependent on TLR4 is unknown.

The observed *in vivo* effects of TLR4 deficiency on cancer cachexia may involve other organs in addition to muscle and immune system. TLR4 is expressed and mediates inflammatory response in many types of cells. In addition, TLR4-mediated elevation of inflammatory cytokines exerts detrimental effects on various organs. For example, they can stimulate lipolysis in adipocytes and cause loss of fat tissue, which frequently takes place in cancer cachexia^34^. The product of excessive lipolysis, free fatty acids, could in turn activate TLR4^35^ to enhance muscle wasting. Further, TLR4 mediates insulin resistance^36, 37^, which is another feature of cancer cachexia. Therefore, TLR4 activation is likely to exert extensive pathological impact on various organs and thus a key contributor to the etiology of cancer cachexia.

In addition to cancer cachexia, TLR4 may mediate cachexia associated with other diseases. We have previously demonstrated that TLR4 mediates lipopolysaccharide (LPS)-induced muscle wasting^38^. In addition, TLR4 plays important roles in the etiology of diabetes and nephropathy^39^, thus, may contribute to cachexia associated with these diseases. Taken together, we showed in the present study a central role of TLR4 in mediating cancer-induced muscle wasting by activating muscle protein degradation pathways, which opens the door for combating cachexia by targeting a single plasma membrane receptor.

## Methods

### Cell cultures

Murine C2C12 myoblasts (American Type Culture Collection) were cultured in growth medium (DMEM supplemented with 10% fetal bovine serum) at 37 °C with 5% CO_2_. At 85–90% confluence, myoblast differentiation was induced by incubation for 96 h in differentiation medium (DMEM supplemented with 4% heat-inactivated horse serum) to form myotubes. Preconditioned medium from cultures of Lewis lung carcinoma cells (obtained from National Institute of Cancer, Frederick, MD) or non-tumorigenic human lung epithelial cell line NL20 (obtained from ATCC) for control were collected from 48 h cultures and centrifuged, the supernatant was used to treat C2C12 myotubes (25% final volume in fresh medium) when indicated. The conditioned medium was replaced with fresh one every 24 h. All cell culture experiments were independently replicated 3 times as indicated (N = 3).

### Animal use

Experimental protocols were approved in advance by the institutional Animal Welfare Committee at the University of Texas Health Science Center at Houston. All methods were performed in accordance with the relevant guidelines and regulations. For LLC-induced cancer cachexia model, 100 μl LLC cells (5 × 10^1^), or an equal volume of PBS (control) was injected subcutaneously into the right flanks of 7-week-old male C57BL/6 (Jackson Laboratories, Bar Harbor, ME) or TLR4^−/−^ mice in the C57BL/6 background (a gift from Dr. S. Akira of Osaka University, Osaka, Japan)^26^. Development of cachexia was monitored by tumor size, body weight and grip strength. Mice were euthanized on day 21 of LLC implant. Tibialis anterior (TA) and extensor digitorum longus (EDL) muscles were then collected immediately for analyses. Each experimental group contained 6 mice (N = 6). To monitor autophagosome formation the TA of wild type C57BL/6 or TLR4^−/−^ mice that had been implanted with LLC cells (or injected with PBS as control) were transfected with GFP-LC3 or GFP-expressing plasmid^40^ (100 μg in 25 μl PBS) on day 14 as previously described^41^. In 7 days, when cachexia was developed frozen cross sections (10 μm) of TA were prepared from euthanized mice with anti-fade mounting solution containing DAPI.

### Transfection of siRNA

Predesigned siRNAs specific for TLR2 and TLR4 were purchased form Sigma-Aldrich (IDS: SASI_Mm01_00135214 and SASI_Mm01_00139037, respectively). Control siRNA was purchased from ThermoFisher Scientific. The siRNAs were introduced into C2C12 myoblasts using the jetPRIME reagent (Polyplus-transfection Inc., Illkirch, France) according to the manufacturer’s protocol. In 24 h, myoblasts were differentiated and experiments were started in another 96 h when myotubes were formed.

### Western blot analysis

Western blot analysis was carried out as described previously^10^. Antibodies to total and/or phosphorylated p38MAPK (T181/Y182) as well as p-C/EBPβ (Thr-188) were from Cell Signaling Technology (Beverly, MA). Antibody to total C/EBPβ, TLR2, TLR4, phosphorylated and total p65 were obtained from Santa Cruz Biotechnology (Santa Cruz, CA). Antibody to atrogin1/MAFbx was from ECM Biosciences (Versailles, KY). Antibodies to UBR2 and LC3-II were obtained from Novus Biologicals (Littleton, CO). Anti-MHC antibody (MF-20) was from R&D Systems (Minneapolis, MN). Data was normalized to α-Tubulin (antibody was from Development Studies Hybridoma Bank at the University of Iowa, Iowa City, IA) or GAPDH (antibody was from Millipore, Billerica, MA).

### Fluorescence microscopy and histology study

C2C12 myotubes were stained with anti-MHC antibody (MF-20) and FITC-conjugated secondary antibody, and examined using a Zeiss Axioskop 40 microscope and a Zeiss Axiocam MRM camera system controlled by Axiovision Release 4.6 imaging software. Acquired images were analyzed for myotube diameter using the method of Menconi *et al*.^42^ with modifications. Briefly, myotube diameters were measured in a total of 100 myotubes from ≥10 random fields using computerized image analysis (Scion Image, Frederick, MD, USA) at 3 points along their length. The average of diameters at the 3 points was used for each myotube. The measurements were conducted in a blind fashion. Cross-sectional area of H&E stained muscle sections was quantified by using the ImageJ software (National Institute of Health). Frozen sections of mouse TA that expressed GFP or LC3-GFP were examined with fluorescence microscopy as described above for C2C12 myotubes.

### Tyrosine release assay

Tyrosine release was measured as described previously^8^.

### *In vitro* TLR4 activation assay

HEK-Blue™-hTLR4 cells (InvivoGen, San Diego, CA) are HEK293 cells co-transfected with the human TLR4, MD-2 and CD14 co-receptor genes, and an inducible SEAP (secreted embryonic alkaline phosphatase) reporter gene that is under the control of NF-κB and AP-1. HEK-Blue-hTLR4 cells and wild type HEK293 cells (as control) were treated with LCM for 24 h. SEAP activity in culture media was measured by using QUANTI-Blue as chromogenic substrate at 630 nm in Synergy 2 Multi-Mode Microplate Reader (Biotek Instruments) following manufacturer’s protocol.

### Bio-Plex^®^ Multiplex Immunoassay

Sera from LLC tumor-bearing mice were analyzed for specific cytokines utilizing Bio-Plex Pro™ Mouse Cytokine Th17 Panel A 6-Plex Group l (Bio-Rad Laboratories, Hercules, CA) according to manufacturer’s protocol. Cytokine concentrations were determined using the Bio-Plex 200 reader (software version 6.0, Bio-Rad Laboratories).

### Statistical analysis

Data were presented as the mean ± S.D., and analyzed by the SigmaStat software (Systat Software, San Jose, CA) with Student t test (data with two groups) or one-way ANOVA (data with more than two groups) followed by a multiple comparison test chosen by the software. Chi-square analysis was carried out by using R to compare the distributions of muscle fiber cross-sectional area among various groups. A *P* value < 0.05 was considered to be statistically significant.
